# Minimal Impact of COVID-19 on One-Year Mortality on US Patients with Cancer Diagnosed Early during the Pandemic

**DOI:** 10.1158/2767-9764.CRC-25-0286

**Published:** 2025-09-15

**Authors:** Anne-Michelle Noone, Nadia Howlader, Angela B. Mariotto, Yoon Duk Hong, Ruth M. Pfeiffer

**Affiliations:** 1 Surveillance Research Program, Division of Cancer Control and Population Sciences, National Cancer Institute, Rockville, Maryland.; 2 Biostatistics Branch, Division of Cancer Epidemiology and Genetics, National Cancer Institute, Rockville, Maryland.

## Abstract

Patients with cancer diagnosed early in the COVID-19 pandemic may have been particularly vulnerable due to immunosuppression caused by their cancer. The objective was to assess cause of death due to COVID-19, cancer, and other causes among patients with cancer during the pandemic. We thus examined causes of death (cancer, COVID-19, other causes) among patients with cancer diagnosed in 2020 (*N* = 503,128) compared with 2018 (*N* = 537,006) in the Surveillance, Epidemiology, and End Results-22. We focused on 1-year cause-specific mortality by cancer type, stage, and age at diagnosis. In a counterfactual analysis, we estimated the number and proportions of patients with cancer dying of cancer or other competing causes if COVID-19 were eliminated as a possible cause of death. Among 2020 patients with cancer, 15.8% died of cancer, 3.3% from other causes, and 0.7% from COVID-19. Among those who died, COVID-19 mortality was 9%, 7.6%, 2.8%, 2.8%, and 1.2% of all deaths for prostate, breast, colorectal, lung, and pancreatic cancers, respectively. In a counterfactual analysis assuming that COVID-19 had not occurred, similar percentages would have died of cancer or other causes, except for lung and pancreatic cancers. In summary, cancer was the leading cause of death in 2020, but COVID-19 did not have a large influence on competing-cause mortality. These findings inform healthcare strategies to mitigate excess mortality among patients with cancer during future healthcare disruptions.

**Significance:**

These findings show that, among patients with cancer, cancer remains a leading cause of death during the COVID-19 pandemic. These results underscore the importance of cancer care even amid other public health emergencies.

## Introduction

People living with cancer have faced a twofold greater risk of dying of COVID-19, caused by severe acute respiratory syndrome coronavirus 2 (SARS-CoV-2), than the general US population (1). Patients with cancer diagnosed during the first year of the pandemic in 2020, when COVID-19 vaccination and therapeutic treatments were not available, may have been especially susceptible to COVID-19 fatality (2, 3). This analysis aims to assess underlying causes of death due to COVID-19, cancer, and other causes among patients with cancer diagnosed during 2020 and characterize the 1-year cause-specific mortality for these competing causes based on patient characteristics such as sex, cancer type, age, and stage at diagnosis.

Whereas the acute phase of the COVID-19 pandemic has passed, understanding its impact on patients with cancer remains highly relevant for several reasons. First, it highlights the potential role of emerging infectious diseases on immunosuppressed populations, offering valuable lessons for future pandemic preparedness and public health planning. Second, this study contributes to the broader understanding of competing-cause mortality, which remains an essential concept in cancer prognosis and survivorship research, irrespective of the pandemic context. Finally, this study utilizes statistical modeling to estimate the burden of cancer during the pandemic had COVID-19 been a possible cause of death (COD). Unlike most prior studies that have either focused exclusively on cancer mortality or cancer deaths and noncancer deaths, our analysis distinguishes between three causes of death: cancer, COVID-19, and other causes of death, allowing for a more nuanced understanding of the competing risks faced by patients with cancer during the pandemic.

We compared the characteristics and 1-year competing risk survival of the patients, including three causes of death: cancer, COVID-19, and other causes of death. In a counterfactual analysis, we estimated the number and proportions of patients with cancer dying of cancer or other competing causes if COVID-19 were eliminated as a possible COD among the patients diagnosed in 2020, i.e., had these patients been diagnosed in 2018.

## Materials and Methods

We included patients with cancer diagnosed at ages 20+ with a first primary cancer in 2018 and in 2020 in the Surveillance, Epidemiology, and End Results (SEER)-22 registries excluding Illinois and Massachusetts, covering approximately 44% of the US population (4, 5). We excluded patients diagnosed by autopsy or death certificate only (*n* = 10,327 for 2018 and *n* = 9,363 for 2020). For this analysis, we used all cancers combined and separately analyzed cancers for five sites. Several cancers were chosen, some with screening recommendations (female breast, prostate, and colorectal cancers), cancers detected mainly by symptoms [lung and bronchus (although there are lung cancer screening guidelines, most lung cancers are still detected based on symptoms)], and pancreas cancer, which has low survival. Cancers with screening guidelines were selected because cancer incidence rates declined substantially in 2020 especially for screen-detected cancers (6). Age at diagnosis was grouped into the categories 20 to 59, 60 to 74, and 75+. Cancer stage was categorized as *in situ*/localized, regional, or distant using the combined Summary Stage 2004+ (7). Underlying causes of death were classified as cancer, COVID-19, and other causes. SEER receives COD information from death certificates obtained by the National Center for Health Statistics. For this analysis, we used the SEER cause-specific death classification variable to classify death from cancer and other causes (8, 9), and the SEER COD site recode variable (10) was used to classify death from COVID-19 (code U07.1) for the 2020 patient cohort. Patients with missing or unknown COD (*n* = 4,306) and those alive but without survival time (*n* = 4,470) were excluded, resulting in a final analysis cohort comprised of *N* = 503,128 patients diagnosed in 2020 and *N* = 537,006 in 2018.

We followed the patients for up to 12 months after cancer diagnosis to assess the 1-year probabilities of death from cancer, COVID-19, and causes other than COVID-19. Patients diagnosed in 2018 were followed through December 31, 2019, and patients diagnosed in 2020 through December 31, 2021.

To obtain the counterfactual event counts for individuals diagnosed in 2020 had they had the experience of those diagnosed in 2018, we predicted their 1-year risk of cancer keeping their predictors X from an outcome model estimated for the 2018 cohort, in the absence of COVID. To be specific, let $D_{i}=1$ if person *i* dies from cancer within 1 year of diagnosis, and $D_{i}=0$ otherwise. Then $D_{i}$ has a binomial distribution with $P\left(D_{i}=1|X_{i}\right)$, in which $X_{i}$ denotes the personal and clinical characteristics of the person. For *N* individuals, the expected number of cancer deaths (CD) within 1 year from diagnosis is then given by $\;\hat{CD}=\overset{N}{\underset{i}{\sum }}P\left(D_{i}=1|X_{i}\right).$ We modeled $P\left(D_{i}=1|X_{i}\right)$ using the absolute risk or cumulative incidence of dying of cancer (cause 1) within 1 year of diagnosis for a person with predictors $X_{i}$ using a cause-specific formulation (11).

$$r\left(X\right)=\int_{0}^{1}\lambda^{1}\left(u|X\right)\exp\left[-\int_{0}^{u}\left\{\lambda^{1}\left(s|X\right)+\lambda^{2}\left(s|X\right)\right\}ds\right]du$$ (A)

In equation (A), $\lambda^{1}\left(u|X\right)$ is the cause-specific hazard for cancer death, and $\lambda^{2}\left(s|X\right)$ is the hazard for death from other causes.

Each hazard was modeled using a Cox proportional hazards regression model as

$$\lambda^{k}\left(t|X\right)=\lambda_{0}^{k}\left(t\right)\exp\left(X^{T}{\text{β}}_{k}\right),k=1,2$$ (B)

and combined in equation (A) based on the R package *riskRegression* (12, 13). To obtain the counterfactual event counts for individuals diagnosed in 2020 had they had the experience of those diagnosed in 2018, we predicted their 1 year risk of cancer keeping their predictors X from the model estimated for the 2018 cohort, in the absence of COVID. We compared the expected number of deaths from cancer for other causes, $\hat{CD}$, for patients diagnosed in 2020 with the observed numbers, O, and calculated the percent change in deaths as (*O-*$\hat{CD}$*)/*$\hat{CD}$)). We also scaled this number up to represent the difference in number of deaths for the whole United States. The scaling factor was the inverse of proportion of the population covered by the SEER areas included in the analysis to the total US population in 2020. The assumption underlying this counterfactual calculation is that there are no unmeasured variables that differ in prevalence and correlation with measured variables between the 2018 and 2020 cohorts that affect the hazards of death of a specific cause. All calculations were conducted in R. This study used publicly available, deidentified data and was not considered human subjects research under the NIH policy; Institutional Review Board review was not required.

### Data availability

This article contains analysis of the Incidence – SEER Research Plus Limited-Field Data, 22 Registries (excluding Illinois and Massachusetts), Nov 2023 Sub (2000–2021). These data are available at https://seer.cancer.gov/data/access.html.

## Results

The distribution of demographic characteristics was similar for the 537,459 patients diagnosed in 2018 and the 503,545 patients diagnosed in 2020 (Table 1). An exception is that those diagnosed in 2020 were more likely to be in the highest income category. Specifically, 28.3% of those diagnosed in 2020 were in the highest income category (≥$95,000) vs. 25.1% of those diagnosed in 2018. Among those diagnosed in 2020, patients with lung and pancreas cancer tended to be older with about one thirds of the 2020 cohort age 75+, whereas less than one quarters of those with breast, prostate, or colorectal cancer were age 75+ at diagnosis. The majority of patients with lung and pancreatic cancers were diagnosed with distant-stage disease, whereas those diagnosed with other cancers tended to be diagnosed with localized or regional disease.

**Table 1.** Characteristics of patients diagnosed in 2018 compared with patients diagnosed in 2020 for all cancer sites combined and for selected cancer sites.

Diagnosis year	All cancer sites				Female breast				Prostate			
	2018		2020		2018		2020		2018		2020	
Number of persons	537,459		503,545		83,233		77,332		79,925		74,073	
	*N*	%	*N*	%	*N*	%	*N*	%	*N*	%	*N*	%
Age at diagnosis, years	​	​	​	​	​	​	​	​	​	​	​	​
<60	181,010	33.7%	164,216	32.6%	37,069	44.5%	35,013	45.3%	16,213	20.3%	13,649	18.4%
60–74	236,751	44.1%	225,348	44.8%	32,791	39.4%	30,186	39.0%	48,960	61.3%	45,905	62.0%
75+	119,698	22.3%	113,981	22.6%	13,373	16.1%	12,133	15.7%	14,752	18.5%	14,519	19.6%
Sex	​	​	​	​	​	​	​	​	​	​	​	​
Female	264,912	49.3%	247,666	49.2%	83,233	100.0%	77,332	100.0%	​	​	​	​
Male	272,547	50.7%	255,879	50.8%	​	​	​	​	79,925	100.0%	74,073	100.0%
Race	​	​	​	​	​	​	​	​	​	​	​	​
NH White	351,708	65.4%	324,540	64.5%	52,536	63.1%	47,850	61.9%	51,041	63.9%	46,907	63.3%
NH Black	60,779	11.3%	56,839	11.3%	9,530	11.4%	9,119	11.8%	13,388	16.8%	12,300	16.6%
NH API or NH AI/AN	38,539	7.2%	36,582	7.3%	7,666	9.2%	7,165	9.3%	3,860	4.8%	3,588	4.8%
Hispanic	79,973	14.9%	77,576	15.4%	12,868	15.5%	12,508	16.2%	9,656	12.1%	8,896	12.0%
Unknown	6,460	1.2%	8,008	1.6%	633	0.8%	690	0.9%	1,980	2.5%	2,382	3.2%
Stage^a^	​	​	​	​	​	​	​	​	​	​	​	​
*In situ*/localized	254,050	47.3%	232,813	46.2%	53,195	63.9%	49,060	63.4%	55,772	69.8%	51,184	69.1%
Regional	111,182	20.7%	107,201	21.3%	22,785	27.4%	21,650	28.0%	10,577	13.2%	10,193	13.8%
Distant	133,080	24.8%	131,048	26.0%	4,982	6.0%	5,041	6.5%	6,377	8.0%	6,768	9.1%
Unknown/unstaged	39,147	7.3%	32,483	6.5%	2,271	2.7%	1,581	2.0%	7,199	9.0%	5,928	8.0%
Rural/urban	​	​	​	​	​	​	​	​	​	​	​	​
Urban	472,744	88.0%	442,069	87.8%	74,707	89.8%	69,325	89.6%	70,607	88.3%	65,648	88.6%
Rural	64,262	12.0%	61,059	12.1%	8,464	10.2%	7,968	10.3%	9,274	11.6%	8,392	11.3%
Unknown	​	0.0%	​	0.0%	62	0.1%	39	0.1%	44	0.1%	33	0.0%
Median household income^b^	​	​	​	​	​	​	​	​	​	​	​	​
<$40K–$59,999	81,842	15.2%	68,654	13.6%	11,185	13.4%	9,391	12.1%	11,901	14.9%	9,468	12.8%
$60K–$79,999	190,440	35.4%	169,098	33.6%	28,757	34.5%	25,345	32.8%	28,170	35.2%	24,482	33.1%
$80K–94,999	130,042	24.2%	123,359	24.5%	20,844	25.0%	19,589	25.3%	19,262	24.1%	18,076	24.4%
≥$95,000	135,095	25.1%	142,420	28.3%	22,441	27.0%	23,005	29.7%	20,579	25.7%	22,043	29.8%
Unknown	40	0.01%	14	0.00%	6	0.01%	2	0.00%	13	0.02%	4	0.01%

Diagnosis year	Lung				Colorectum				Pancreas			
	2018		2020		2018		2020		2018		2020	
Number of persons	55,303		50,223		45,420		41,658		15,852		16,043	
	*N*	%	*N*	%	*N*	%	*N*	%	*N*	%	*N*	%
Age	​	​	​	​	​	​	​	​	​	​	​	​
<60	9,974	18.0%	8,142	16.2%	16,719	36.8%	15,704	37.7%	3,570	22.5%	3,328	20.7%
60–74	27,697	50.1%	25,970	51.7%	17,608	38.8%	15,960	38.3%	7,288	46.0%	7,527	46.9%
75+	17,632	31.9%	16,111	32.1%	11,093	24.4%	9,994	24.0%	4,994	31.5%	5,188	32.3%
Sex	​	​	​	​	​	​	​	​	​	​	​	​
Female	27,225	49.2%	24,680	49.1%	21,109	46.5%	19,400	46.6%	7,595	47.9%	7,845	48.9%
Male	28,078	50.8%	25,543	50.9%	24,311	53.5%	22,258	53.4%	8,257	52.1%	8,198	51.1%
Race	​	​	​	​	​	​	​	​	​	​	​	​
NH White	40,112	72.5%	36,327	72.3%	27,688	61.0%	25,156	60.4%	10,230	64.5%	10,196	63.6%
NH Black	6,247	11.3%	5,588	11.1%	5,612	12.4%	5,025	12.1%	1,994	12.6%	2,040	12.7%
NH API or NH AI/AN	4,025	7.3%	3,764	7.5%	3,839	8.5%	3,608	8.7%	1,176	7.4%	1,214	7.6%
Hispanic	4,778	8.6%	4,358	8.7%	7,884	17.4%	7,456	17.9%	2,425	15.3%	2,543	15.9%
Unknown	141	0.3%	186	0.4%	397	0.9%	413	1.0%	27	0.2%	50	0.3%
Stage^a^	​	​	​	​	​	​	​	​	​	​	​	​
*In situ*/localized	13,402	24.2%	11,705	23.3%	15,780	34.7%	13,309	31.9%	2,396	15.1%	2,517	15.7%
Regional	10,992	19.9%	9,864	19.6%	16,159	35.6%	15,713	37.7%	4,480	28.3%	4,410	27.5%
Distant	28,393	51.3%	26,591	52.9%	10,551	23.2%	10,438	25.1%	8,005	50.5%	8,335	52.0%
Unknown/unstaged	2,516	4.5%	2,063	4.1%	2,930	6.5%	2,198	5.3%	971	6.1%	781	4.9%
Rural/urban	​	​	​	​	​	​	​	​	​	​	​	​
Urban	46,616	84.3%	42,032	83.7%	39,497	87.0%	36,188	79.7%	13,949	30.7%	14,137	31.1%
Rural	8,628	15.6%	8,134	16.2%	5,851	12.9%	5,397	11.9%	1,889	4.2%	1,886	4.2%
Unknown	59	0.1%	57	0.1%	72	0.2%	73	0.2%	14	0.0%	20	0.0%
Median household income^b^	​	​	​	​	​	​	​	​	​	​	​	​
<$40K–$59,999	10,435	18.9%	8,602	17.1%	7,784	17.1%	6,308	15.1%	2,414	15.2%	2,189	13.6%
$60K–$79,999	20,427	36.9%	18,091	36.0%	15,886	35.0%	13,812	33.2%	5,608	35.4%	5,376	33.5%
$80K–$94,999	12,281	22.2%	11,115	22.1%	11,018	24.3%	10,345	24.8%	3,891	24.5%	3,993	24.9%
≥$95,000	12,160	22.0%	12,415	24.7%	10,731	23.6%	11,192	26.9%	3,939	24.8%	4,485	28.0%
Unknown	0	0%	0	0%	1	0.0%	1	0.0%	0	0.0%	0	0.0%

Abbreviations: AI/AN, American Indian/Alaska Native; API, Asian/Pacific Islander; NH, non-Hispanic.

a Combined Summary Stage 2004+.

b Median household income is a county-level variable (https://seer.cancer.gov/seerstat/variables/countyattribs/).

Among patients diagnosed with all cancers combined in 2020, 79,286 (15.8%) died of cancer within 1 year of diagnosis, 16,705 (3.3%) died of other competing causes, and 3,577 died (0.7%) of COVID-19 (Table 2). By the end of the one-year follow-up, 403,560 patients remained alive (80.2%). The distribution of outcomes within 1 year from diagnosis was similar for the 537,006 patients diagnosed in 2018 (i.e., the prepandemic cohort); 15.2% died of cancer, 3.1% died of other causes, and 81.7% remained alive. Among patients diagnosed in 2020 who died, the percentage of deaths due to COVID-19 was the highest for patients with prostate cancer (9%) and breast cancer (7.6%) and lower for those with colorectal cancer (2.8%), lung cancer (2.8%), and pancreatic cancer (1.2%).

**Table 2.** Observed outcomes within 1 year of cancer diagnosis among patients diagnosed in 2018 and 2020 and expected outcomes for those diagnosed in 2020 had they been diagnosed in 2018, i.e., had COVID-19 not occurred.

Outcome	2018 cohort		2020 cohort		2020 cohort in the absence of COVID		Observed in 2020–expected in 2020		
	Observed outcomes within 1 year of diagnosis		Observed outcomes within 1 year of diagnosis		Expected deaths within 1 year of diagnosis		SEER	US population	Change in % total observed–expected in 2020
	*N*	% Total	*N*	% Total	*N*	% Total	*N*	*N*	%
All cancer sites combined	​	​	​	​	​	​	​	​	​
Alive	438,472	81.7%	403,560	80.2%	407,901	81.1%	−4,341	−10,356	−0.9%
Died of cancer	81,872	15.2%	79,286	15.8%	79,600	15.8%	−314	−750	−0.1%
Died of other causes	16,662	3.1%	16,705	3.3%	15,627	3.1%	1,078	2,572	0.2%
Died of COVID	0	​	3,577	0.7%	0	​	3,577	8,533	0.7%
Female breast	​	​	​	​	​	​	​	​	​
Alive	80,072	96.3%	74,119	95.9%	74,394	96.2%	−275	−657	−0.4%
Died of cancer	2,246	2.7%	2,149	2.8%	2,130	2.8%	19	46	0.0%
Died of other causes	853	1.0%	785	1.0%	769	1.0%	16	39	0.0%
Died of COVID	0	​	240	0.3%	0	0.0%	240	573	0.3%
Prostate	​	​	​	​	​	​	​	​	​
Alive	77,385	96.9%	71,160	96.1%	71,548	96.6%	−388	−926	−0.5%
Died of cancer	1,448	1.8%	1,497	2.0%	1,489	2.0%	8	20	0.0%
Died of other causes	1,048	1.3%	1,124	1.5%	1,003	1.4%	121	289	0.2%
Died of COVID	0	​	259	0.3%	0	​	259	618	0.3%
Colorectum	​	​	​	​	​	​	​	​	​
Alive	37,451	82.6%	33,393	80.3%	34,151	82.1%	−758	−1809	−1.8%
Died of cancer	6,424	14.2%	6,412	15.4%	6,107	14.7%	305	728	0.7%
Died of other causes	1,473	3.2%	1,501	3.6%	1,327	3.2%	174	416	0.4%
Died of COVID	0	​	279	0.7%	0	0.0%	279	666	0.7%
Lung	​	​	​	​	​	​	​	​	​
Alive	29,712	53.8%	26,705	53.2%	26,058	51.9%	647	1,544	1.3%
Died of cancer	22,176	40.1%	19,691	39.3%	21,081	42.0%	−1,390	−3,316	−2.8%
Died of other causes	3,356	6.1%	3,118	6.2%	3,027	6.0%	91	218	0.2%
Died of COVID	0	​	652	1.3%	0	0.0%	652	1,556	1.3%
Pancreas	​	​	​	​	​	​	​	​	​
Alive	6,263	39.5%	6,235	38.9%	5,944	37.1%	291	695	1.8%
Died of cancer	9,004	56.9%	9,032	56.4%	9,521	59.4%	−489	−1,167	−3.1%
Died of other causes	571	3.6%	643	4.0%	558	3.5%	85	203	0.5%
Died of COVID	0	​	113	0.7%	0	0.0%	113	270	0.7%

**Figure 1.**
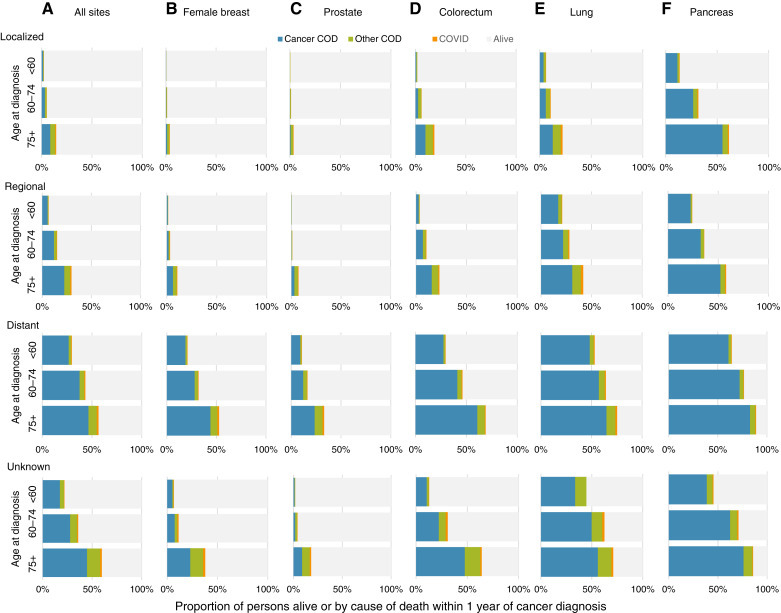
**A–F,** Proportion of persons experiencing each outcome within 1 year after cancer diagnosis among those diagnosed in 2020 for all cancer sites (**A**), female breast cancer (**B**), prostate cancer (**C**), colorectal cancer (**D**), lung cancer (**E**), and pancreatic cancer (**F**). Data are shown in Table 3.

Figure 1A–F illustrates the empirical probabilities of surviving, dying of cancer, competing causes of death, and COVID-19 within 1-year for 2020 diagnosis for all cancers combined and by five cancer types stratified by age at diagnosis and stage. The most influential factor on survival was stage (Fig. 1A–F; Table 3). Across all cancer sites and ages, those diagnosed with distant stage disease had poorer survival compared with those in local and regional stages. Moreover, the primary COD especially among those with later-stage disease was cancer. For example, among women ages 60 to 74-years, the probability of dying of breast cancer was 0.3% when diagnosed with localized disease, 1.8% for regional stage, and 28.1% for distant stage; for lung cancer, the corresponding probabilities were 5.8% for patients diagnosed with localized disease, 21.8% for regional stage, and 57.3% for distant disease.

**Table 3.** Number of persons alive and by COD within 1 year of cancer diagnosis among patients diagnosed in 2020.

Cancer site	Stage^a^	Age at diagnosis	Died of cancer		Died of COVID-19		Died of other causes		Alive	
All cancer sites combined	*In situ*/localized	20–59	1,160	1.5%	103	0.1%	444	0.6%	77,311	97.8%
		60–74	3,416	3.2%	402	0.4%	1,741	1.6%	102,533	94.9%
		75+	3,870	8.5%	444	1.0%	2,244	4.9%	38,990	85.6%
	Regional	20–59	2,251	5.4%	79	0.2%	387	0.9%	38,648	93.4%
		60–74	5,465	11.9%	292	0.6%	1,216	2.7%	38,904	84.8%
		75+	4,461	22.5%	265	1.3%	1,201	6.0%	13,937	70.2%
	Distant	20–59	9,636	26.6%	272	0.7%	904	2.5%	25,461	70.2%
		60–74	21,737	37.5%	667	1.2%	2,718	4.7%	32,799	56.6%
		75+	17,057	46.5%	654	1.8%	3,050	8.3%	15,940	43.4%
	Unknown	20–59	1,283	17.3%	40	0.5%	302	4.1%	5,776	78.0%
		60–74	3,672	27.7%	151	1.1%	938	7.1%	8,511	64.1%
		75+	5,278	44.7%	208	1.8%	1,560	13.2%	4,750	40.3%
Female breast	*In situ*/localized	20–59	30	0.1%	12	0.1%	36	0.2%	20,239	99.6%
		60–74	65	0.3%	53	0.3%	134	0.6%	20,598	98.8%
		75+	96	1.2%	39	0.5%	167	2.1%	7,565	96.2%
	Regional	20–59	116	1.0%	14	0.1%	34	0.3%	11,755	98.6%
		60–74	122	1.8%	39	0.6%	66	1.0%	6,701	96.7%
		75+	172	6.2%	22	0.8%	100	3.6%	2,501	89.5%
	Distant	20–59	408	>18%	Na	​	37	1.7%	1,718	79.1%
		60–74	529	28.1%	13	0.7%	62	3.3%	1,281	68.0%
		75+	431	44.0%	18	1.8%	68	6.9%	463	47.2%
	Unknown	20–59	30	>5%	Na	​	Na	​	544	>92%
		60–74	38	7.5%	Na	​	13	2.6%	451	>88%
		75+	112	23.0%	11	2.3%	62	12.7%	303	62.1%
Prostate	*In situ*/localized	20–59	Na	​	Na	​	31	<0.5%	9,725	>99%
		60–74	52	0.2%	73	0.2%	249	0.8%	32,141	98.8%
		75+	66	0.7%	49	0.6%	211	2.4%	8,557	96.3%
	Regional	20–59	Na	​	Na	​	Na	​	2,203	>99%
		60–74	14	0.2%	10	0.1%	36	0.5%	6,754	99.1%
		75+	34	2.9%	Na	​	41	3.5%	1,079	>92%
	Distant	20–59	77	9.0%	Na	​	11	1.3%	764	>89%
		60–74	382	12.0%	20	0.6%	119	3.7%	2,666	83.7%
		75+	640	23.5%	45	1.7%	215	7.9%	1,820	66.9%
	Unknown	20–59	9	1.1%	Na	​	Na	​	794	>98%
		60–74	62	1.8%	14	0.4%	64	1.9%	3,226	95.8%
		75+	155	8.9%	27	1.5%	136	7.8%	1,431	81.8%
Lung	*In situ*/localized	20–59	52	3.6%	Na	<0.5%	30	2.1%	1,350	93.9%
		60–74	362	5.8%	40	0.6%	260	4.2%	5,555	89.4%
		75+	507	12.5%	69	1.7%	317	7.8%	3,147	77.9%
	Regional	20–59	275	17.0%	10	0.6%	53	3.3%	1,278	79.1%
		60–74	1,183	21.8%	66	1.2%	272	5.0%	3,898	71.9%
		75+	869	30.8%	67	2.4%	241	8.6%	1,640	58.2%
	Distant	20–59	2,344	48.3%	51	1.1%	187	3.9%	2,272	46.8%
		60–74	7,698	57.3%	160	1.2%	781	5.8%	4,803	35.7%
		75+	5,351	64.8%	145	1.8%	733	8.9%	2,032	24.6%
	Unknown	20–59	76	33.6%	0	0.0%	25	11.1%	125	55.3%
		60–74	430	49.8%	19	2.2%	90	10.4%	324	37.5%
		75+	544	55.9%	19	2.0%	129	13.3%	281	28.9%
Colorectum	*In situ*/localized	20–59	37	0.8%	Na	​	47	1.0%	4,816	>98%
		60–74	135	2.6%	20	0.4%	164	3.1%	4,909	93.9%
		75+	313	9.9%	47	1.5%	232	7.4%	2,556	81.2%
	Regional	20–59	155	2.6%	14	0.2%	51	0.8%	5,806	96.3%
		60–74	398	6.7%	41	0.7%	173	2.9%	5,328	89.7%
		75+	578	15.5%	40	1.1%	243	6.5%	2,863	76.9%
	Distant	20–59	1,135	27.5%	21	0.5%	68	1.6%	2,907	70.4%
		60–74	1,665	41.1%	39	1.0%	167	4.1%	2,179	53.8%
		75+	1,358	60.8%	19	0.9%	164	7.3%	693	31.0%
	Unknown	20–59	63	10.4%	Na	​	12	2.0%	527	>87%
		60–74	159	22.2%	15	2.1%	48	6.7%	495	69.0%
		75+	416	47.5%	13	1.5%	132	15.1%	314	35.9%
Pancreas	*In situ*/localized	20–59	65	11.4%	Na	​	10	1.8%	493	86.3%
		60–74	282	26.7%	11	1.0%	42	4.0%	720	68.2%
		75+	491	55.2%	10	1.1%	46	5.2%	343	38.5%
	Regional	20–59	205	<23%	Na	​	12	1.3%	692	>76%
		60–74	725	<34%	Na	​	71	3.2%	1,424	>63%
		75+	660	52.2%	15	1.2%	57	4.5%	532	42.1%
	Distant	20–59	1,059	60.9%	11	0.6%	47	2.7%	623	35.8%
		60–74	2,851	72.1%	22	0.6%	155	3.9%	925	23.4%
		75+	2,176	82.8%	21	0.8%	141	5.4%	290	11.0%
	Unknown	20–59	39	>38%	Na	​	Na	​	55	>54%
		60–74	174	>62%	Na	​	20	>7%	81	<30%
		75+	305	>76%	Na	​	36	<10%	57	>14%

Na: Case counts less than 10 were suppressed for confidentiality; ranges are given for percentages so that cause counts cannot be backcalculated.

a Combined Summary Stage 2004+.

Within each stage, the overall probability of death increased with older ages. For example, the survival probability decreased from 98.6% for 20- to 59-year-old patients with breast cancer with regional disease to 89.5% for 75+-year-old women. In addition, within each stage, the chance of dying of other causes also increased with age; for example, men diagnosed between ages 20- to 59-year-old with distant disease had a 1.3% probability of dying of other causes; however, 75+-year-old men diagnosed with the same disease had a 7.9% probability of death due to other causes.

The probabilities of COVID-19 death among patients with cancer varied little by cancer type, age at diagnosis, and stage. For patients with breast cancer, the probabilities of death from COVID-19 were low, ranging from 0.1% to 2.3%. Similarly, for lung cancer patients, the chances of death from COVID-19 ranged from 0.4% to 2.2%, depending on age and stage.

In the counterfactual analysis, we compared the deaths observed in 2020 compared with what the same patients with cancer would have experienced had they been diagnosed in 2018. Overall in the United States, the cohort of people diagnosed with cancer in 2020 experienced 750 fewer deaths from cancer, 2,572 more deaths from other causes, and 10,356 fewer individuals survived than if they had been diagnosed before the pandemic (Table 1). These observed deaths represent less than a 1% change from what was expected for each COD. Patients with breast and prostate cancers experienced almost no change in the observed number of deaths attributed to cancer or other causes compared with what was expected in the absence of COVID-19. They did experience slightly more deaths than expected (*N* = 657 for breast and 926 for prostate for the whole United States). Among patients diagnosed with colorectal cancer, 728 more deaths from cancer and 416 more deaths from other causes were observed in 2020 compared with what was expected in the absence of COVID-19. Patients diagnosed with lung or pancreatic cancer experienced slightly more deaths from other causes. However, they experienced fewer deaths from cancer compared with what was expected in the absence of COVID-19 (*N* = 3,316 for lung and *N* = 1,167 for pancreas for the whole United States).

## Discussion

In summary, among patients with cancer diagnosed in 2020, we observed 99,568 deaths within 1 year of cancer diagnosis. Among those who died, cancer remained the leading COD (15.8%), whereas COVID-19 deaths were rare (0.7%). Older age increased the risk of dying of other competing causes, especially for those with cancer in localized or regional stages. Whereas stage at diagnosis had the strongest impact on survival, COVID-19 mortality varied little by age, stage, or cancer type. Under a counterfactual analysis estimating 2020 mortality outcomes as if patients were diagnosed in 2018 (i.e., without COVID-19), we found that similar percentages would have died of cancer or other causes, except for patients with lung and pancreatic cancers, who were more likely to die of other causes during and less likely to die of cancer during the COVID-19 pandemic. Hong and colleagues (2) have shown that 1-year survival was lower for patients diagnosed in Q2 of 2020 but that survival in Q3 and Q4 was similar to prepandemic levels. These results support those conclusions as we analyzed all patients diagnosed within the year. Furthermore, Brar and colleagues (14) found that those with cancer and COVID-19 infection had similar mortality to those without cancer, supporting our finding that COD remains fairly consistent over time.

A limitation of this study is that COD may be misattributed on the death certificate. Indeed, previous work has found that deaths due to COVID-19 may be undercounted or unrecognized especially early in the pandemic and among those ages 65 and older (15, 16). We found more deaths from other causes and fewer cancer deaths during the pandemic than expected among those with pancreatic and lung cancers. These patients were diagnosed at older ages and with more advanced cancer and may have had additional comorbid conditions, making it difficult to correctly identify the COD. We were only able to assess 1 year of follow-up after a cancer diagnosis in 2020 due to availability of data. For cancers with high 1-year survival, the impact of treatment delays or lack of access to medical care may not be observed until a longer follow-up period becomes available.

Strengths of this study include using high-quality population-based data from the SEER-22 cancer registries and accounting for differences in the covariate distributions between diagnosis years when estimating the counterfactual experience of those diagnosed in 2020 without COVID-19.

Despite the COVID-19 pandemic creating disruptions in cancer treatment, we observed that the overall number of deaths 1 year after diagnosis was not greatly affected among recently diagnosed patients with cancer. However, this study reinforces the need for continued follow-up beyond 1 year to help assess the pandemic’s evolving impact on cancer outcomes more comprehensively. Our findings also provide insight into the resilience of cancer care and mortality patterns during the pandemic and potentially during future public health crises. They also underscore the importance of maintaining access to timely diagnosis and treatment in future emergencies.

## References

[1] Mani KA , Wu X, Spratt DE, Wang M, Zaorsky NG. A population-based study of COVID-19 mortality risk in US cancer patients. J Natl Cancer Inst 2024;116:1288–93. 38621700 10.1093/jnci/djae086

[2] Hong YD , Howlader N, Noone A-M, Mariotto AB. Assessing the effect of the COVID-19 pandemic on 1-year cancer survival in the United States. J Natl Cancer Inst 2025;117:1064–8. 39453989 10.1093/jnci/djae271 PMC12058259

[3] Wang Q , Berger NA, Xu R. Analyses of risk, racial disparity, and outcomes among US patients with cancer and COVID-19 infection. JAMA Oncol 2021;7:220–7. 33300956 10.1001/jamaoncol.2020.6178 PMC7729584

[4] SEER . SEER*Stat software. Bethedsa (MD): National Cancer Institute. [cited 2024 Nov 14]. Available from: https://seer.cancer.gov/seerstat/.

[5] SEER . SEER*Stat Database: incidence - SEER research plus limited-field data, 22 registries (excl IL and MA), Nov 2023 Sub (2000-2021) - linked to county attributes - time dependent (1990-2022) income/rurality, 1969-2022 counties, National Cancer Institute, DCCPS, Surveillance Research Program, released April 2024, based on the November 2023 submission. Bethedsa (MD): National Cancer Institute.

[6] Howlader N , Bhattacharya M, Scoppa S, Miller D, Noone A-M, Negoita S, . Cancer and COVID-19: US cancer incidence rates during the first year of the pandemic. J Natl Cancer Inst 2024;116:208–15. 37796818 10.1093/jnci/djad205 PMC10852612

[7] SEER . Localized/regional/distant stage adjustment. Bethedsa (MD): National Cancer Institute. [cited 2024 Nov 14]. Available from: https://seer.cancer.gov/seerstat/variables/seer/lrd-stage/.

[8] Howlader N , Ries LA, Mariotto AB, Reichman ME, Ruhl J, Cronin KA. Improved estimates of cancer-specific survival rates from population-based data. J Natl Cancer Inst 2010;102:1584–98. 20937991 10.1093/jnci/djq366 PMC2957430

[9] SEER . SEER cause-specific death classification. Bethedsa (MD): National Cancer Institute. [cited 2024 Nov 14]. Available from: https://seer.cancer.gov/causespecific/.

[10] SEER . SEER cause of death recode 2024. Bethedsa (MD): National Cancer Institute. [cited 2024 Nov 14]. Available from: https://seer.cancer.gov/codrecode/.

[11] Pfeiffer RM , Gail MH. Absolute risk: methods and applications in clinical management and public health. Taylor & Francis; 2018.

[12] Gerds TA , Ohlendorff JS, Blanche P, Mortensen R, Wright M, Tollenaar N, . riskRegression: risk regression models and prediction scores for survival analysis with competing risks [internet]. 2023 [cited 2024 Nov 14]. Available from: https://CRAN.R-project.org/package=riskRegression.

[13] Gerds TA , Kattan MW. Medical risk prediction models: with ties to machine learning. 1st ed.. Chapman and Hall/CRC; 2021.

[14] Brar G , Pinheiro LC, Shusterman M, Swed B, Reshetnyak E, Soroka O, . COVID-19 severity and outcomes in patients with cancer: a matched cohort study. J Clin Oncol 2020;38:3914–24. 32986528 10.1200/JCO.20.01580 PMC7676890

[15] Chen Y-H , Stokes AC, Aschmann HE, Chen R, DeVost S, Kiang MV, . Excess natural-cause deaths in California by cause and setting: march 2020 through February 2021. PNAS Nexus 2022;1:pgac079. 35832865 10.1093/pnasnexus/pgac079 PMC9272175

[16] Iuliano AD , Chang HH, Patel NN, Threlkel R, Kniss K, Reich J, . Estimating under-recognized COVID-19 deaths, United States, march 2020-may 2021 using an excess mortality modelling approach. Lancet Reg Health Am 2021;1:100019. 34386789 10.1016/j.lana.2021.100019 PMC8275579

